# Risk management of cardiovascular disease in older patients with diabetes

**DOI:** 10.1038/s41440-025-02424-4

**Published:** 2025-10-16

**Authors:** Kosuke Sawami, Atsushi Tanaka, Koichi Node

**Affiliations:** 1 Department of Frontier Cardiovascular Science, The University of Tokyo Hospital, Tokyo, Japan; 2 Department of Cardiovascular Medicine, Saga University, Saga, Japan

**Keywords:** Type 2 diabetes, Older adults, Glucagon-like peptide-1 receptor agonist, Sodium-glucose cotransporter 2 inhibitor, Implemental hypertension

## Abstract

The number of older patients with diabetes has been increasing due to Japan’s rapidly aging population. As aging and diabetes are independent risk factors for cardiovascular disease, older adults face a higher risk of cardiovascular disease, with the incidence of heart failure and stroke increasing significantly with age. Preventing cardiovascular events is critical for this population to maintain their activities of daily living and improve their quality of life. To achieve this challenging purpose, it is essential to prioritize the use of glucose-lowering medications with proven cardiovascular benefits and to comprehensively manage risk factors, including blood pressure, as with younger patients. However, considering the vulnerability and divergent health status of older patients, population-specific effects and precautions regarding medications, such as sodium-glucose cotransporter (SGLT) 2 inhibitors and glucagon-like peptide 1 receptor agonists (GLP-1RA) should be considered. In particular, we must deliberate their impact on frailty when administering these medications to this population. In this mini review, we discuss the characteristics of older patients with diabetes and update the evidence on the consistent efficacy and safety of SGLT2 inhibitors and GLP-1RA in older patients. In addition, recent clinical trials that examined the effect of stricter blood pressure control in older patients with diabetes have been reconsidered to accomplish comprehensive risk management for preventing cardiovascular disease.

**Figure:**
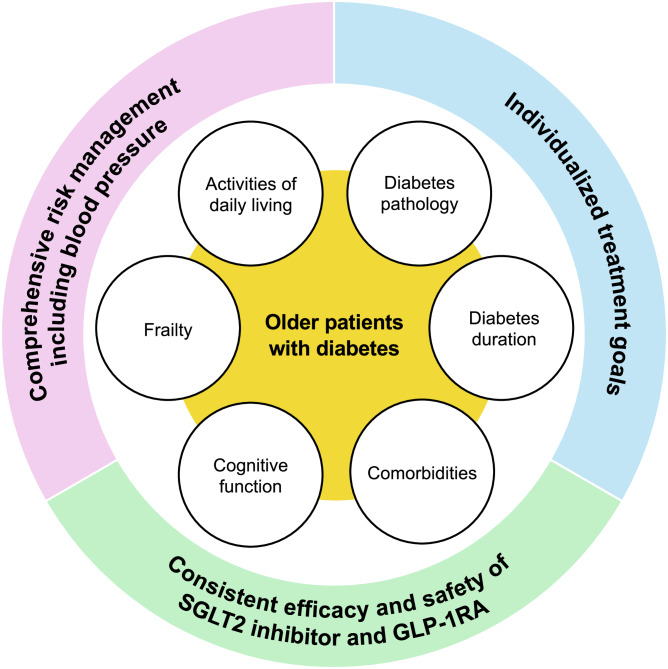
Heterogeneity of older patients with diabetes and principles of cardiovascular risk management in this population. *SGLT2:* sodium glucose cotransporter 2, *GLP-1RA:* glucagon-like peptide 1 receptor agonist

Heterogeneity of older patients with diabetes and principles of cardiovascular risk management in this population. *SGLT2:* sodium glucose cotransporter 2, *GLP-1RA:* glucagon-like peptide 1 receptor agonist

## Abstract (web summary)

Heterogeneity of older patients with diabetes and principles of cardiovascular risk management in this population. SGLT2: sodium glucose cotransporter 2, GLP-1RA: glucagon-like peptide 1 receptor agonist

## Diabetes in older adults

As both diabetes and aging are major independent risk factors for cardiovascular disease, older patients with these conditions are considered the highest-risk group for cardiovascular disease. According to the National Health and Nutrition Survey in 2023, among all age groups, the highest proportion of people aged 70 years or older reported an HbA1c level of 6.5% or higher or were under treatment for diabetes (26.2% of men and 13.7% of women) [1]. In a registry study including 72,310 patients with type 2 diabetes aged 60 years or older in the United States, the most common complication was cardiovascular disease (coronary artery disease, stroke and heart failure) in both groups diagnosed with diabetes less than 10 years prior and more than 10 years prior [2]. Regardless of the duration of diabetes, the incidence of cardiovascular disease was 2–3 times higher in those aged 80 years or older than in those in their 60 s, with heart failure and stroke showing a particularly sharp increase with age [2]. Limitations in physical activities and dyspnea due to heart failure and post-stroke sequelae such as paralysis, dysphagia and aphasia significantly impair activities of daily living (ADL), exacerbate geriatric syndrome and reduce life expectancy [3]. Therefore, cardiovascular events must be prevented to avoid ADL decline and maintain quality of life (QOL) in older patients with diabetes, especially in Asian patients who have higher incidence of stroke [4]. Additionally, while both microvascular complications and cardiovascular disease prone to occur more frequently with longer diabetes duration, the impact of diabetes duration on cardiovascular disease is less severe than that on microvascular complications [2]. In other words, comprehensive management focused on preventing cardiovascular disease is important, regardless of the duration of diabetes.

However, older patients with diabetes constitute a highly diverse population, varying not only in diabetes pathophysiology and duration but also in comorbidities, cognitive function, frailty and ADL decline. Establishing individualized treatment goals that consider these factors is critical [3, 5]. The American Diabetes Association (ADA) Standards of Care in Diabetes 2025 recommends dividing older patients into three categories and setting individualized management goals corresponding to each category [5]. In the “Healthy” group, in which patients have few comorbidities, preserved cognitive and physical function, and are expected to have long-term prognosis, treatment goals for blood glucose, blood pressure and lipids could be equivalent to those of younger individuals through shared decision-making between patients and healthcare providers. On the other hand, for the “Very complex/poor health” group, which is in the palliative or end-of-life care phase or has severe cognitive impairment, the primary goal for blood glucose management is to avoid hypoglycemia and hyperglycemic emergencies. In the “Complex/intermediate” group, which falls between these two groups, patients have multiple comorbidities and two or more ADL impairments or moderate decline in cognitive function. This group is the most diverse in terms of life expectancy, severity of comorbidities, and cognitive and physical functions. As life expectancy decreases, the benefits derived from lowering blood glucose levels are considered to diminish [5]. Hence, a continuous assessment of the balance between the benefits and adverse effects of treatments for diabetes and its complications including cardiovascular disease is required. Selecting glucose-lowering medications with cardiovascular protective effects and implementing comprehensive risk management including blood pressure could contribute to the prevention of cardiovascular events, maintenance of ADL and improvement in QOL.

Collectively, cardiovascular risk management in older patients with diabetes should be based on careful consideration of individual patient characteristics with the following principles: (1) prioritizing and continuing the use of glucose-lowering agents that also have favorable cardiovascular effects as long as possible, and (2) paying sufficient attention to the management of other cardiovascular risk factors, especially blood pressure control.

## Selection of glucose-lowering medication with cardioprotective effect in older adults

Sodium-glucose cotransporter (SGLT) 2 inhibitors and glucagon-like peptide 1 receptor agonists (GLP-1RA) are prominent glucose-lowering agents that have been confirmed to reduce cardiovascular events in patients with type 2 diabetes at high cardiovascular risk. The ADA Standards of Care in Diabetes 2025 recommends the inclusion of medications with proven cardiovascular benefits in treatment plans for older patients with diabetes who have prior cardiovascular disease or multiple cardiovascular risks regardless of blood glucose levels [5]. This section summarizes the effects of SGLT2 inhibitors and GLP-1RA on older patients with type 2 diabetes and the considerations for their prescription to older patients (Table 1).

**Table 1:** Characteristics of SGLT2 inhibitors and GLP-1RA regarding administration for older patients with diabetes

	SGLT2 inhibitor	GLP-1RA
Representative medications approved in Japan	Empagliflozin Dapagliflozin Canagliflozin	Semaglutide (oral and subcutaneous) Liraglutide (subcutaneous) Dulaglutide (subcutaneous) Lixisenatide (subcutaneous)
Major effect on cardiovascular disease	In patients with type2 diabetes at high cardiovascular risk, MACE: approximately 10% ↓ HHF: approximately 30% ↓	In patients with type2 diabetes at high cardiovascular risk, MACE: approximately 14% ↓
Consistency in efficacy on older patients with diabetes	MACE and HHF ↓	MACE ↓
Characteristics on formulation	Less likely to cause hypoglycemia when used alone All agents are once-daily oral formulations	Less likely to cause hypoglycemia when used alone Strong glucose-lowering effect Oral or subcutaneous injection Once-daily or once-weekly formulation
Caution in administration to older patients	Dehydration Genital infection Malnutrition	Excessive weight loss Gastrointestinal symptoms

*SGLT2* sodium glucose cotransporter 2 inhibitor, *GLP-1RA* glucagon-like peptide 1 receptor agonist, *MACE* major adverse cardiovascular events, *HHF* hospitalization for heart failure

SGLT2 inhibitors have been shown to reduce major adverse cardiovascular events (MACE, a composite endpoint of myocardial infarction, stroke and cardiovascular death) and hospitalization for heart failure in large clinical trials in patients with type 2 diabetes at high cardiovascular risk. A meta-analysis including EMPA-REG OUTCOME (empagliflozin), CANVAS Program (canagliflozin), DECLARE-TIMI 58 (dapagliflozin), CREDENCE (canagliflozin) and VERTIS CV (ertugliflozin) demonstrated that SGLT2 inhibitors reduced MACE by 10% (hazard ratio [HR] 0.90, 95% confidence interval [CI] 0.85–0.95) and hospitalization for heart failure by 32% (HR 0.68, 95% CI 0.61–0.76) compared to placebo [6].

In the age-stratified subgroup analysis of EMPA-REG OUTCOME, the effect of empagliflozin on MACE was demonstrated by HRs of 0.86 (95% CI 0.74–0.99) overall, 1.04 (95% CI 0.84–1.29) in those under 65 years, 0.74 (95% CI 0.58–0.93) in those aged 65–74 years, and 0.68 (95% CI 0.46–1.00) in those aged 75 years or older, indicating a trend toward greater efficacy with increasing age (*p*_interaction_ = 0.047) [7]. For hospitalization for heart failure, the overall HR was 0.65 (95% CI 0.50–0.85), while the HR was 0.73 (95% CI 0.48–1.10) for those under 65 years of age, 0.66 (95% CI 0.44–1.00) for those aged 65–74 years, and 0.45 (95% CI 0.22–0.89) for those aged 75 years or older. However, no interaction was observed (*p*_interaction_ = 0.488) [7].

In a meta-analysis that stratified the results of the five clinical trials by age at 65 or 75 years [8], the HR for MACE was 0.94 (95% CI 0.56–1.03) in those under 65 years and 0.87 (95% CI 0.74–1.01) in those aged 65 years or older (*p*_interaction_ = 0.38), 0.93 (95%CI 0.85–1.02) in those under 75 years and 0.77 (95% CI 0.60–0.99) in those aged 75 years or older (*p*_interaction_ = 0.16). These results suggest that the effect of SGLT2 inhibitors on MACE is consistent even in older patients. Regarding hospitalization for heart failure, the HR was 0.83 (95% CI 0.67–1.04) for those under 65 years of age and 0.62 (95% CI 0.51–0.76) for those aged 65 years or older (*p*_interaction_ = 0.06), 0.72 (95% CI 0.61–0.84) for those under 75 years and 0.64 (95% CI 0.36–1.12) for those aged 75 years or older (*p*_interaction_ = 0.70), with no evidence of reduced efficacy in older patients. Although previous clinical trials suggest that SGLT2 inhibitors have a neutral effect on stroke [6], the HR was 1.18 (95% CI 0.94–1.48) in those under 65 years of age and 0.83 (95% CI 0.69–1.00) in those aged 65 years or older (*p*_interaction_ = 0.02), suggesting a different efficacy on stroke between older and younger patients. Overall, SGLT2 inhibitors could be administered to older patients with type 2 diabetes to reduce the incident of MACE and hospitalization for heart failure.

SGLT2 inhibitors are known to have an insulin-independent hypoglycemic effect and are less likely to cause hypoglycemia when used as monotherapy. These agents are once-daily oral formulations, making them more accessible to older patients. However, adverse effects such as dehydration, genital infections and malnutrition are concerned especially when these medications are administered to older patients [3]. While SGLT2 inhibitors often result in a weight loss of approximately 2–3 kg, no reduction in muscle mass or bone mineral density was observed with empagliflozin administration in the EMPA-ELDERLY study [9], which evaluated the effects of empagliflozin on glucose levels and muscle mass in Japanese patients with type 2 diabetes aged 65 years or older with a body mass index (BMI) of 22 kg/m² or higher. While patients in frail experienced higher incidence of genital infections when they received SGLT2 inhibitors than those receiving DPP-4 inhibitors, no increases in the incidence of hypoglycemia, ketoacidosis, lower limb amputation and fractures were observed regardless of the frail status in a cohort study of 744,310 patients with type 2 diabetes aged 65 years or older [10]. Furthermore, the absolute risk reduction of SGLT2 inhibitors for the composite outcome of hospitalization due to myocardial infarction, stroke or heart failure, and all-cause mortality was higher in patients with frailty. In other words, even in older patients with frailty, reducing the incidence of MACE and hospitalization for heart failure may be possible by continuing SGLT2 inhibitors with careful follow-up.

GLP-1RA have also demonstrated cardioprotective effects in patients with type 2 diabetes at high cardiovascular risk. A meta-analysis of eight clinical trials including ELIXA (lixisenatide), LEADER (liraglutide), SUSTAIN-6 (subcutaneous injectable semaglutide), EXSCEL (exenatide), Harmony Outcomes (albiglutide), REWIND (duraglutide), PIONEER-6 (oral administrated semaglutide) and AMPLITUDE-O (efpeglenatide) showed that GLP-1RA reduced MACE by 14% (HR 0.86, 95% CI 0.80–0.93) [11]. In addition, GLP-1RA have more pronounced efficacy on MACE for Asian patients with diabetes [12]. Given that GLP-1RA reduce the risk of stroke by 17% (HR 0.83, 95% CI 0.76–0.92) [11], GLP-1RA could play a significant role on reducing cardiovascular events in Asian population.

In a post-hoc analysis of the Harmony Outcome [13], the HR on MACE was 0.77 (95% CI 0.66–0.89) overall, 0.66 (95% CI 0.53–0.82) in those under 65 years and 0.86 (95% CI 0.71–1.04) in those aged 65 years or older (*p*_interaction_ = 0.07), 0.78 (95% CI 0.67–0.91) in those under 75 years and 0.70 (95% CI 0.48–1.01) in those aged 75 years or older (*p*_interaction_ = 0.60). Therefore, the effect of albiglutide on MACE was not influenced by age.

In an age-stratified meta-analysis of ELIXA, LEADER, SUSTAIN-6, EXSCEL, REWIND and PIONEER-6, the HR on MACE was 0.89 (95% CI 0.76–1.03) in those under 65 years of age and 0.86 (95% CI 0.80–0.92) in those aged 65 years or older (*p*_interaction_ = 0.73), 0.92 (95% CI 0.85–0.99) in those under 75 years and 0.75 (95% CI 0.61–0.92) in those 75 years or older (*p*_interaction_ = 0.07), indicating that the efficacy of GLP-1RA for MACE is consistent in older patients [8].

GLP-1RA are less likely to cause hypoglycemia when used alone, while having a higher glucose-lowering effect [14]. Therefore, this could be a strategy to address polypharmacy in cases where multiple glucose-lowering agents can be consolidated into a GLP-1RA. Semaglutide is available in injectable (Ozempic^R^) or oral (Rybelsus^R^) formulations, whereas liraglutide, lixisenatide and dulaglutide only have injectable formulations. Although oral semaglutide does not require injection, its oral administration method is complicated. Therefore, careful consideration is necessary when administering it to older patients with declined cognitive function. As semaglutide and dulaglutide are administered as once-weekly subcutaneous injection formulations, they potentially improve adherence by decreasing the number of oral medications and reduce the burden on caregivers.

Although excessive weight loss caused by GLP-1RA may exacerbate frailty in older patients, 24 weeks of daily administration of 3 mg liraglutide resulted in reduced fat mass without decreasing muscle mass in older patients with type 2 diabetes and obesity [15]. Moreover, GLP-1RAs may inhibit muscle atrophy by increasing blood flow and inducing metabolic changes in skeletal muscle [16]. Given the fact that gastrointestinal symptoms are more common in older patients, careful consideration is required when using GLP-1RAs in older patients with diabetes having a lean body composition.

## Blood pressure management in older patients with diabetes

Management of hypertension is potent for reducing cardiovascular events also in older adults [17], however, the target blood pressure for patients with diabetes should be individualized through shared decision-making between the patient and healthcare provider, taking into account cardiovascular risks and adverse events [18]. The Japanese Society of Hypertension Guidelines for the Management of Elevated Blood Pressure and Hypertension 2025 (JSH2025) recommends blood pressure less than 130/80 mmHg regardless of age or comorbidities [19]. It includes older patients over 75 years of age who can independently visit clinics and maintain ADL. For older patients with declined instrumental ADL and requiring help for clinic visits, or with declined basic ADL and difficulty in visiting clinics, target blood pressure should be discussed considering comorbidities based on systolic blood pressure less than 140 mmHg or 150 mmHg, respectively. The ADA Standards of Care in Diabetes 2025 also recommends maintaining blood pressure less than 130/80 mmHg in patients with diabetes when it is considered safely achieved [18]. Although lowering systolic blood pressure to less than 130 mmHg reduces the risk of stroke, it remains unclear whether it reduces the risk of coronary artery disease, cardiovascular mortality or all-cause mortality according to the results from clinical trials of patients with diabetes and hypertension [20, 21].

The ADA and some guidelines in Europe state that the target blood pressure for patients with diabetes should not be set below 120/70 mmHg [18, 22, 23], based on the results of the ACCORD study, which evaluated whether maintaining systolic blood pressure less than 120 mmHg reduces cardiovascular events in patients with type 2 diabetes at high cardiovascular risk. In the ACCORD study [24], systolic blood pressure reached an average of 119.3 mmHg in the intensive treatment group at the 1 year follow-up. Over an average observation period of 4.7 years, there was no statistically significant reduction in MACE compared to the standard treatment group targeting less than 140 mmHg (HR 0.88, 95% CI 0.73–1.06), while the incidence stroke was reduced in the intensive treatment group (HR 0.59, 95% CI 0.39–0.89). Whereas the result of ACCORD must be interpreted carefully because it might have been influenced by the lack of statistical power for assessing cardiovascular events and the effects of baseline characteristics like statin or aspirin use, most of current guidelines generally do not recommend maintaining systolic blood pressure less than 120 mmHg to reduce mortality or cardiovascular disease other than stroke considering adverse events related to antihypertensive therapy, such as worsening renal function and electrolyte abnormalities.

The SPRINT study showed a reduction in the composite of myocardial infarction, acute coronary syndrome, stroke, acute heart failure and cardiovascular death by lowering systolic blood pressure to less than 120 mmHg in patients with hypertension at high cardiovascular risk [25]. Subsequently, evidence is accumulating in favor of strict blood pressure control targeting less than 120 mmHg in populations including older patients with diabetes.

In the STEP trial [26], 8511 patients with hypertension aged 60–80 years were randomized to the intensive treatment group targeting systolic blood pressure of 110–130 mmHg and the standard treatment group targeting 130–150 mmHg. Among the participants, 19.1% had diabetes, and 24% were aged 70–80 years. At the 1 year follow-up, the average systolic blood pressure was 127.5 mmHg in the intensive treatment group and 135.3 mmHg in the standard treatment group. At a median follow-up of 3.34 years, the incident of the composite endpoint of stroke, acute coronary syndrome, acute heart failure, coronary revascularization, atrial fibrillation and cardiovascular death was lower in the intensive treatment group (HR 0.71, 95% CI 0.56–0.91), in addition to the lower incidence of stroke (HR 0.67, 95% CI 0.47–0.97), acute coronary syndrome (HR 0.67, 95% CI 0.47–0.94) and acute heart failure (HR 0.27, 95% CI 0.08–0.98). Moreover, the effect of strict blood pressure control on the composite endpoint was consistent regardless of age (60–69 years and 70–80 years) and the presence of diabetes.

In the ESPRIT trial [27], 11,255 patients with hypertension aged 50 years or older with a high cardiovascular risk were randomized to the intensive treatment group targeting a systolic blood pressure less than 120 mmHg or the standard treatment group targeting less than 140 mmHg. Among the patients, 38.7% had diabetes, and 24% were aged 70 years or older. At a median follow-up of 3.4 years, the mean systolic blood pressure was 119.1 mmHg in the intensive treatment group, 134.8 mmHg in the standard treatment group. Incidence of the composite endpoint of myocardial infarction, coronary revascularization, hospitalization for heart failure, stroke or cardiovascular death was lower in the intensive treatment group (HR 0.88, 95% CI 0.78–0.99), with no interaction observed based on diabetes status or age. While cardiovascular mortality (HR 0.61, 95% CI 0.44–0.84) and all-cause mortality (HR 0.79, 95% CI 0.64–0.97) decreased, no statistically significant differences were observed in the incidence of myocardial infarction and stroke.

The BPROAD study [28] included 12,821 patients with type 2 diabetes and hypertension aged 50 years or older, and 48% were aged above 65 years. Patients were randomized into the intensive treatment group targeting a systolic blood pressure less than 120 mmHg and the standard treatment group targeting less than 140 mmHg. At the 1 year follow-up, the mean systolic blood pressure was 121.6 mmHg in the intensive treatment group and 133.2 mmHg in the standard treatment group. At a median follow-up of 4.2 years, incidence of the composite endpoint of stroke, acute coronary syndrome, heart failure and cardiovascular death was lower in the intensive treatment group (HR 0.79, 95% CI 0.69–0.90), with no age-related interaction observed.

**Fig. 1:**
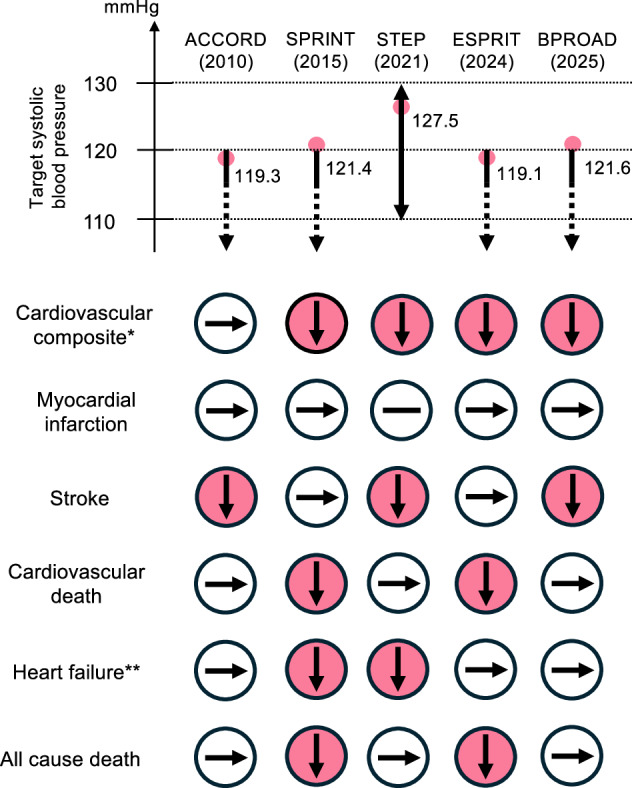
Comparison of target systolic blood pressure and outcomes of major clinical trials. Upper panel shows target systolic blood pressure for each trial and pink dots represent mean achieved systolic blood pressure. Lower panel shows the trial outcomes. * Major adverse cardiovascular events (MACE) in ACCORD; MACE, acute coronary syndrome (ACS), or hospitalization or urgent visit for heart failure in SPRINT; stroke, ACS, coronary revascularization, atrial fibrillation, hospitalization or urgent visit for heart failure, or cardiovascular death in STEP; myocardial infarction (MI), coronary or non-coronary revascularization, hospitalization or emergency visits for new-onset heart failure or acute decompensated heart failure, stroke, or cardiovascular death in ESPRIT; stroke, MI, treatment or hospitalization for heart failure, or cardiovascular death in BPROAD. ** Death or hospitalization for heart failure (HF) in ACCORD; hospitalization or emergency visits for HF in other trials

These findings suggest that strict systolic blood pressure management achieving less than 120 mmHg may further reduce cardiovascular events in populations including elderly patients with diabetes (Fig. 1), and the latest guideline on hypertension from the American College of Cardiology and the American Heart Association mentions the target less than 120 mmHg in patients with diabetes [29]. However, caution is warranted as higher rates of adverse events such as symptomatic hypotension, syncope and electrolyte abnormalities were reported in the intensive treatment group. Generally, the intensification of blood pressure management and occurrence of these adverse events represent a trade-off, therefore, assessing individual tolerance is important.

## Summary

Older patients with diabetes are also at a high risk of cardiovascular disease. The risk of heart failure and stroke especially increases rapidly with age and significantly affects ADL and QOL. SGLT2 inhibitors are effective in preventing MACE and hospitalization for heart failure, and GLP-1RAs also have comparable effects on MACE consistently in older patients with diabetes at a high cardiovascular risk. Careful follow-up may allow the administration of these agents and reduce the incidence of MACE and hospitalization for heart failure even in patients with frailty.

Individualized blood pressure control is an indispensable component of cardiovascular risk management in older patients with diabetes. Major guidelines recommend blood pressure less than 130/80 mmHg in patients with diabetes including older patients. Recent clinical trials such as STEP, ESPRIT and BPROAD have suggested that strictly maintaining systolic blood pressure less than 120 mmHg may reduce cardiovascular events. However, intensive blood pressure lowering may increase adverse events such as hypotension and electrolyte abnormalities, therefore, decisions should be made considering the patient’s individual tolerance.

## References

[1] Ministry of Health, Labor and Welfare. The National Health and Nutrition Survey in Japan, 2023. https://www.mhlw.go.jp/stf/seisakunitsuite/bunya/kenkou_iryou/kenkou/eiyou/r5-houkoku_00001.html. Accessed 4 August 2025.

[2] Huang ES, Laiteerapong N, Liu JY, John PM, Moffet HH, Karter AJ. Rates of complications and mortality in older patients with diabetes mellitus: the diabetes and aging study. JAMA Intern Med. 2014;174:251–8. 24322595 10.1001/jamainternmed.2013.12956 PMC3950338

[3] The Japan Geriatrics Society, The Japan Diabetes Society. Practice Guideline for the Treatment of Elderly Diabetes. Nankodo: Tokyo, Japan; 2023.

[4] Woodward M, Patel A, Zoungas S, Liu L, Pan C, Poulter N, et al. Does glycemic control offer similar benefits among patients with diabetes in different regions of the world? Results from the ADVANCE trial. Diabetes Care. 2011;34:2491–5. 21972410 10.2337/dc11-0755 PMC3220831

[5] American Diabetes Association Professional Practice C. 13. Older adults: standards of care in diabetes-2025. Diabetes Care. 2025;48:S266–S82. 10.2337/dc25-S013 PMC11635042 39651977

[6] McGuire DK, Shih WJ, Cosentino F, Charbonnel B, Cherney DZI, Dagogo-Jack S, et al. Association of SGLT2 inhibitors with cardiovascular and kidney outcomes in patients with type 2 diabetes: a meta-analysis. JAMA Cardiol. 2021;6:148–58. 33031522 10.1001/jamacardio.2020.4511 PMC7542529

[7] Monteiro P, Bergenstal RM, Toural E, Inzucchi SE, Zinman B, Hantel S, et al. Efficacy and safety of empagliflozin in older patients in the EMPA-REG OUTCOME(R) trial. Age Ageing. 2019;48:859–66. 31579904 10.1093/ageing/afz096 PMC7963112

[8] Karagiannis T, Tsapas A, Athanasiadou E, Avgerinos I, Liakos A, Matthews DR, et al. GLP-1 receptor agonists and SGLT2 inhibitors for older people with type 2 diabetes: a systematic review and meta-analysis. Diabetes Res Clin Pract. 2021;174:108737. 33705820 10.1016/j.diabres.2021.108737

[9] Yabe D, Shiki K, Homma G, Meinicke T, Ogura Y, Seino Y, et al. Efficacy and safety of the sodium-glucose co-transporter-2 inhibitor empagliflozin in elderly Japanese adults (>/=65 years) with type 2 diabetes: a randomized, double-blind, placebo-controlled, 52-week clinical trial (EMPA-ELDERLY). Diabetes Obes Metab. 2023;25:3538–48. 37622398 10.1111/dom.15249

[10] Kutz A, Kim DH, Wexler DJ, Liu J, Schneeweiss S, Glynn RJ, et al. Comparative cardiovascular effectiveness and safety of SGLT-2 inhibitors, GLP-1 receptor agonists, and DPP-4 inhibitors according to frailty in type 2 diabetes. Diabetes Care. 2023;46:2004–14. 37677118 10.2337/dc23-0671 PMC10620535

[11] Sattar N, Lee MMY, Kristensen SL, Branch KRH, Del Prato S, Khurmi NS, et al. Cardiovascular, mortality, and kidney outcomes with GLP-1 receptor agonists in patients with type 2 diabetes: a systematic review and meta-analysis of randomised trials. Lancet Diabetes Endocrinol. 2021;9:653–62. 34425083 10.1016/S2213-8587(21)00203-5

[12] Lee MMY, Ghouri N, Misra A, Kang YM, Rutter MK, Gerstein HC, et al. Comparative efficacy of glucagon-like peptide 1 receptor agonists for cardiovascular outcomes in asian versus white populations: systematic review and meta-analysis of randomized trials of populations with or without type 2 diabetes and/or overweight or obesity. Diabetes Care. 2025;48:489–93. 39977628 10.2337/dc24-1533 PMC12081317

[13] Gilbert MP, Skelly J, Hernandez AF, Green JB, Krychtiuk KA, Granger CB, et al. Effect of albiglutide on cardiovascular outcomes in older adults: a post hoc analysis of a randomized controlled trial. Diabetes Obes Metab. 2024;26:1714–22. 38317618 10.1111/dom.15479

[14] Davies MJ, Aroda VR, Collins BS, Gabbay RA, Green J, Maruthur NM, et al. Management of hyperglycemia in type 2 diabetes, 2022. A consensus report by the American Diabetes Association (ADA) and the European Association for the Study of Diabetes (EASD). Diabetes Care. 2022;45:2753–86. 36148880 10.2337/dci22-0034 PMC10008140

[15] Perna S, Guido D, Bologna C, Solerte SB, Guerriero F, Isu A, et al. Liraglutide and obesity in elderly: efficacy in fat loss and safety in order to prevent sarcopenia. A perspective case series study. Aging Clin Exp Res. 2016;28:1251–7. 26749118 10.1007/s40520-015-0525-y

[16] Szekeres Z, Nagy A, Jahner K, Szabados E. Impact of selected glucagon-like peptide-1 receptor agonists on serum lipids, adipose tissue, and muscle metabolism-a narrative review. Int J Mol Sci. 2024;25:8214. 10.3390/ijms25158214 PMC11311305 39125786

[17] Beckett NS, Peters R, Fletcher AE, Staessen JA, Liu L, Dumitrascu D, et al. Treatment of hypertension in patients 80 years of age or older. N Engl J Med. 2008;358:1887–98. 18378519 10.1056/NEJMoa0801369

[18] American Diabetes Association Professional Practice C. 10. Cardiovascular disease and risk management: standards of care in diabetes-2025. Diabetes Care. 2025;48:S207–S38. 10.2337/dc25-S010 PMC11635050 39651970

[19] The Japanese Society of Hypertension. Guidelines for the Management of Elevated Blood Pressure and Hypertension 2025. Life Science Publishing: Tokyo, Japan, (2025).

[20] Sawami K, Tanaka A, Node K. Recent understandings about hypertension management in type 2 diabetes: what are the roles of SGLT2 inhibitor, GLP-1 receptor agonist, and finerenone?. Hypertens Res. 2023;46:1892–9. 37258623 10.1038/s41440-023-01324-9

[21] Abe M, Segawa H, Kinguchi S, Satoh A, Zamami R, Nishikido T, et al. Intensive blood pressure-lowering treatment to prevent cardiovascular events in patients with diabetes: a systematic review and meta-analysis. Hypertens Res. 2025;48:2024–33. 40269228 10.1038/s41440-025-02209-9

[22] Mancia G, Kreutz R, Brunström M, Burnier M, Grassi G, Januszewicz A, et al. 2023 ESH Guidelines for the management of arterial hypertension The Task Force for the management of arterial hypertension of the European Society of Hypertension: Endorsed by the International Society of Hypertension (ISH) and the European Renal Association (ERA). J Hypertens. 2023;41:1874–2071. 37345492 10.1097/HJH.0000000000003480

[23] McEvoy JW, McCarthy CP, Bruno RM, Brouwers S, Canavan MD, Ceconi C, et al. 2024 ESC Guidelines for the management of elevated blood pressure and hypertension. Eur Heart J. 2024;45:3912–4018. 39210715 10.1093/eurheartj/ehae178

[24] Cushman WC, Evans GW, Byington RP, Goff DC Jr, Grimm RH Jr, Cutler JA, et al. Effects of intensive blood-pressure control in type 2 diabetes mellitus. N Engl J Med. 2010;362:1575–85. 20228401 10.1056/NEJMoa1001286 PMC4123215

[25] Group SR, Wright JT Jr, Williamson JD, Whelton PK, Snyder JK, Sink KM, et al. A randomized trial of intensive versus standard blood-pressure control. N Engl J Med. 2015;373:2103–16. 26551272 10.1056/NEJMoa1511939 PMC4689591

[26] Zhang W, Zhang S, Deng Y, Wu S, Ren J, Sun G, et al. Trial of intensive blood-pressure control in older patients with hypertension. N Engl J Med. 2021;385:1268–79. 34491661 10.1056/NEJMoa2111437

[27] Liu J, Li Y, Ge J, Yan X, Zhang H, Zheng X, et al. Lowering systolic blood pressure to less than 120 mm Hg versus less than 140 mm Hg in patients with high cardiovascular risk with and without diabetes or previous stroke: an open-label, blinded-outcome, randomised trial. Lancet. 2024;404:245–55. 38945140 10.1016/S0140-6736(24)01028-6

[28] Bi Y, Li M, Liu Y, Li T, Lu J, Duan P, et al. Intensive blood-pressure control in patients with type 2 diabetes. N Engl J Med. 2025;392:1155–67. 39555827 10.1056/NEJMoa2412006

[29] Jones DW, Ferdinand KC, Taler SJ, Johnson HM, Shimbo D, Abdalla M, et al. 2025 AHA/ACC/AANP/AAPA/ABC/ACCP/ACPM/AGS/AMA/ASPC/NMA/PCNA/SGIM Guideline for the Prevention, Detection, Evaluation, and Management of High Blood Pressure in Adults: A Report of the American College of Cardiology/American Heart Association Joint Committee on Clinical Practice Guidelines. J Am Coll Cardiol. Published online August 14, 2025. 10.1016/j.jacc.2025.05.007 10.1016/j.jacc.2025.05.007 40815242

